# Biochar enhances the growth and physiological characteristics of *Medicago sativa*, *Amaranthus caudatus* and *Zea mays* in saline soils

**DOI:** 10.1186/s12870-024-04957-1

**Published:** 2024-04-22

**Authors:** Ghulam Murtaza, Muhammad Rizwan, Muhammad Usman, Sajjad Hyder, Muhammad Irfan Akram, Maha Deeb, Jawaher Alkahtani, Bandar M. AlMunqedhi, A. S. Hendy, Mohamed R. Ali, Rashid Iqbal, Wiwiek Harsonowati, Muhammed Habib ur Rahman, Muhammad Rizwan

**Affiliations:** 1 Faculty of Environmental Science and Engineering, Kunming University of Science and Technology, Kunming, 650500 China; 2 School of Energy Science and Engineering, Central South University, Changsha, 410083 China; 3 School of Agriculture and Biology, Shanghai Jiao Tong University, 800 Dongchuan Road, Minghang District, Shanghai, 200240 China; 4 Department of Botany, Government College Women University Sialkot, Sialkot, 51310 Pakistan; 5 Department of Entomology, Faculty of Agriculture and Environment, The Islamia University of Bahawalpur, Bahawalpur, 63000 Pakistan; 6 Soils and Substrates, HEPIA, HES-SO University of Applied Sciences and Arts Western Switzerland, Geneva, Switzerland; 7 Department of Botany and Microbiology, College of Science, King Saud University, P. O. 2455, Riyadh, 11451 Saudi Arabia; 8 Department of Computational Mathematics and Computer Science, Institute of Natural Sciences and Mathematics, Ural Federal University, 19 Mira St., Yekaterinburg, 620002 Russia; 9 Faculty of Engineering and Technology, Future University in Egypt, New Cairo, 11835 Egypt; 10 Basic Engineering Science Department, Benha Faculty of Engineering, Benha University, Benha, 13518 Egypt; 11 Department of Agronomy, Faculty of Agriculture and Environment, The Islamia University of Bahawalpur, Bahawalpur, 63100 Pakistan; 12 Research Center for Horticulture, National Research and Innovation Agency (BRIN), Cibinong, 16915 Bogor Indonesia; 13 Institute of Crop Science and Resource Conservation (INRES), University of Bonn, 53115 Bonn, Germany

**Keywords:** Salinity, *Medicago sativa*, *Amaranthus caudatus*, *Zea mays*, Biochar, Plant growth

## Abstract

Biochar is a promising solution to alleviate the negative impacts of salinity stress on agricultural production. Biochar derived from food waste effect was investigated on three plant species, *Medicago sativa*, *Amaranthus caudatus*, and *Zea mays*, under saline environments. The results showed that biochar improved significantly the height by 30%, fresh weight of shoot by 35% and root by 45% of all three species compared to control (saline soil without biochar adding), as well as enhanced their photosynthetic pigments and enzyme activities in soil. This positive effect varied significantly between the 3 plants highlighting the importance of the plant-biochar interactions. Thus, the application of biochar is a promising solution to enhance the growth, root morphology, and physiological characteristics of plants under salt-induced stress.

## Introduction

Salinity stress is continuously increasing globally, and it has been reported that 900 million hectares around the globe are salt-affected [1]. By the end of 2050, half of the arable land will be damaged by salinity stress owing to the continuous accumulation of salts due to fertilization, salty irrigation, and climate change [2]. Salinity stress inhibits the germination, growth, development, and productivity of plants in both dry and irrigated regions [3]. Salinity stress is an intense abiotic stress that negatively affects plant physiological and biochemical processes and leads to a serious reduction in growth and yield [4].

Salinity stress limits germination, growth, and development by inducing osmotic stress and pseudo-drought stress [5]. The increased concentration of salts in the root zone under salinity stress causes metabolic disorders, affects the photosynthetic efficiency, and assimilates production, which resultantly affects plant growth rate [6]. Salinity stress also disrupts the ionic balance in cells and leads to excessive production of reactive oxygen species (ROS), which causes oxidation of crucial molecules such as membranes, lipids, proteins, and carbohydrates, alters redox homeostasis, and hinders the plant growth [7]. Moreover, salinity stress also increases electrolyte leakage and MDA accumulation, and it also disturbs nutrient uptake, thereby reducing plant growth [3]. Therefore, as a matter of global food security, solutions must be put forth to reclaim and treat salt-affected soils to support better plant growth and productivity under salinity stress conditions [2].

Moreover, El Nahhas et al. [8] revealed a reduction in the activity of photosynthesis in sorghum plants cultivated in saline environments, which was attributed to a noticeable loss in both plant development and yield. Moreover, the toxic effects of Na^+^ and Cl^−^ suppressed the growth of roots, stems, leaves, boll (rounded seed-bearing capsule of a cotton or flax plant), and other organs and even led to death, ultimately causing a decrease in crop yield and biomass [6]. The confluence of enhanced intensity and longer duration of salt stress results in a significant agricultural production decrease [9–12]. To alleviate the salinity stress faced in agricultural production, and to improve crop performances, novel management strategies are needed. To mitigate the negative impacts of salt stress, the biochar application is an attractive solution and sustainable technology to restore degraded land resources [4]. Biochar is a carbon-rich, porous, solid substance that is created through a process called thermochemical transformation, typically in conditions with limited oxygen. Biochar is obtained from organic matter pyrolysis with low oxygen. It had several properties such as high porosity and cation exchange capacity, large surface area, and mineral enrichment [11]. It is commonly used as a soil supplement to improve the water retention and nutrient content of soils [12–15]. It is well known that biochar can act as an important growth regulator, a biostimulant of agricultural productivity, and a driver of plant growth under salt stress. Using biochar amendment has been regarded as an effective strategy for mitigating the negative effects of soil salt stress. Biochar improves the growth of lettuce in saline soils [16, 17]. According to Farid et al. [15], biochar effectively alleviated salt stress in rice seedlings. Biochar enhanced the growth and physiological traits of quinoa plants exposed to drought and salt stress, demonstrating its ability to mitigate the negative effects of both stressors. Furthermore, prior research has demonstrated that biochar can supply a significant quantity of potassium (K) to the soil. This, in turn, mitigates the adverse impact of imbalanced K^+^ uptake by plants caused by the high concentration of sodium chloride (NaCl) in saline soils [18, 19]. Therefore, biochar can serve as a viable alternative to chemical potassium fertilizers. However, numerous recent studies have predominantly shown the individual impacts of salt stress, biochar amendment, and various irrigation regimes on different crops. Additionally, the study on biochar has primarily concentrated on non-saline soils [20]. There have been a limited number of researches undertaken to examine the impact of adding biochar to saline soils. It is crucial to examine the effects of various biochars on the growth of crops and their physiological traits in saline soils.

The study examined the impacts of biochar addition on plant nutrient levels and enzyme activities in soil, focusing on different plant species in both stress and normal conditions [5]. Research conducted by Abo-Elyousr et al. [21] found that the use of biochar enhances the anatomical and physiological characteristics of mungbean plants when exposed to salt stress. Therefore, there is an urgent need to find a synergistic approach to solve the current problem of severe soil salinization to meet crop productivity in future climatic situations. *Medicago sativa*, *Amaranthus cruentus*, and *Zea mays* were chosen as plants capable of tolerating saline soil conditions. The impact of biochar on enhancing the flora of saline soil was examined. The current study assessed the positive influence of biochar on the growth-related parameters and physiological traits of *Medicago sativa, Amaranthus cruentus*, and *Zea mays*, as well as the activities of soil enzymes in saline sand environments.

## Materials and methods

### Experimental site description, soil, seed, and biochar

The experimental site is situated in the Islamia University Bahawalpur, located at coordinates 29.3981° N and 71.6908° E, in Bahawalpur, Punjab, Pakistan. Bahawalpur lies on 117 m above sea level. The climate here is dry. During the year, there is virtually no rainfall in Bahawalpur. The average annual temperature is 25.7 °C | 78.3 °F in Bahawalpur. The rainfall here is around 143 mm | 5.6 inches per year. The chief crops are wheat, gram, cotton, sugarcane, and dates. Sheep and cattle are raised for the export of wool and hides. East of Bahawalpur is the Pat, or Bar, a tract of land considerably higher than the adjoining valley. It is mainly desert irrigated by the Sutlej inundation canals and yields crops of wheat, cotton, and sugarcane. Farther east, the Rohi, or Cholistan, is a barren desert tract, bounded on the north and west by the Hakra depression with mound ruins of old settlements along its high banks; nomads inhabit it. The experiment started in October 2022 and ended in July 2023. For the production of food waste biochar, waste feedstock was collected sun-dried in a dust-free environment, and then oven-dried at 65 °C in an air-derived oven until a constant weight was obtained. The dried material was crushed into small pieces (with a size of 5–10 mm) followed by pyrolysis in a muffle furnace at 550 °C. The gradual increase in furnace temperature from the starting room temperature was set at 8 to 9 °C per minute. 20 min of residence time was set after achieving 550 °C. After allowing it to cool at room temperature, biochar was collected from the furnace, and it was then finely ground until it had a particle size of ≤ 2 mm.

Table 1 describes the characteristics of the biochar produced from food biomass.

**Table 1:** The attributes of biochar are created from food waste

Organic carbon (g/kg)	328.8
Organic matter (g/kg)	567
Total phosphorus (g/kg)	5.10
Total nitrogen (g/kg)	0.30
Available nitrogen (mg/kg)	240.1
Total potassium (g/kg)	41.5
Available phosphorus (mg/kg)	0.66
Available potassium (mg/kg)	687.1
pH	7.99

Sand samples were collected from the dry bottom of Satluj River, Bahawalpur. The Satluj River is the lengthiest among the five rivers that traverse the historically significant crossroads area of Punjab in northern India and Pakistan. The Sutlej River is alternatively referred to as Satadru. The Indus River’s easternmost tributary. Tables 2 and 3 display an analysis of the physico-chemical features of sand, as well as the amount of salinity in the sand. The seeds of *Zea mays* (var. DK-6317), *Amaranthus caudatus* (var. Ames-18,027), and *Medicago sativa* (var. Salt king) were taken from the Regional Agricultural Research Institute (RARI) and employed in the field trials.

**Table 2:** Physical and chemical characteristics of sands

Chemical characteristics	Values
Soil organic carbon (g/kg)	0.88
Soil organic matter (g/kg)	1.49
Total phosphorus (g/kg)	0.26
Total nitrogen (g/kg)	0.58
Available nitrogen (mg/kg)	1.31
Total potassium (g/kg)	0.70
Available phosphors (mg/kg)	1.21
Available potassium (mg/kg)	43.2
pH	8.6

**Table 3:** The level of salinity in collected sand samples

Bicarbonate (g/kg)	0.03
Chloride (g/kg)	4.25
Sulfate (g/kg)	11.39
Calcium (g/kg)	2.09
Magnesium (mg/kg)	1.39
Sodium (g/kg)	628.7
Potassium (mg/kg)	42.97
pH	8.6

### Experimental design

The experimental study was carried out on saline sands located in the dry bottom of the Satluj River. The experiment aimed to investigate the biochar impact on the growth parameters, root morphology, and plant physiological characteristics of *Zea mays*, *Amaranthus caudatus*, and *Medicago sativa*. The experiment was conducted at the Islamia University Bahawalpur applying randomised block design with 3 repetitions. The experimental process consisted of the following: A1—*Medicago sativa* (Control), A2—*Medicago sativa* (Biochar), A3—*Amaranthus caudatus* (Control), A4—*Amaranthus caudatus* (Biochar), A5—*Zea mays* (Control), and A6—*Zea mays* (Biochar). Upon harvesting the plants after 40 days, the fresh weight of the roots, shoots, as well as plant height was determined.

### Evaluation of the root parameters

The roots of *Amaranthus caudatus and Medicago sativa* were cleaned with water with extreme caution. The entirety of the root system was dissected using a scanning system, and the results were evaluated with the help of the Win RHIZO program.

### Determination of the physiological characteristics

The spectrophotometric approach of Hiscox and Israelstam [22] was used to determine the photosynthetic pigments of *Zea mays*, *Amaranthus caudatus*, and *Medicago sativa*. Photosynthetic pigments were determined by the modified method of Hiscox and Israelstam [13]. Fresh leaves were collected in the morning. Fifty mg of fine pieces of fresh leaf sample 2 to 3 mm in size were cut and added to test tubes containing 5 mL of DMSO. Then the test tubes were incubated at 37 °C for 4 h in the dark. The incubation was continued until completely colourless tissue was obtained. The absorbance of the extract was taken at 470 nm, 645 nm, and 663 nm using a spectrophotometer against a DMSO blank. The chlorophyll a (Chl a), chlorophyll b (Chl b), total chlorophyll, and carotenoid contents were determined using the following equations:

1 $$\begin{aligned}\text{C}\text{h}\text{l}\text{o}\text{r}\text{o}\text{p}\text{h}\text{y}\text{l}\text{l}\;\text{a}&=\left[\left(12.7\times \text{O}.\text{D} 663\right)-\left(2.69\times \text{O}.\text{D}645\right)\right]\\ &\quad\times \frac{\text{V}}{1000}\times \text{W}\end{aligned}$$

2 $$\begin{aligned}\text{C}\text{h}\text{l}\text{o}\text{r}\text{o}\text{p}\text{h}\text{y}\text{l}\text{l}\;\text{b}&=[\left(22.9\times \text{O}.\text{D}645\right)-\left(4.68\times \text{O}.\text{D}663\right)]\\&\quad\times \text{V}/100\end{aligned}$$

3 $$\begin{aligned}\text{T}\text{o}\text{t}\text{a}\text{l}\;\text{c}\text{h}\text{l}\text{o}\text{r}\text{o}\text{p}\text{h}\text{y}\text{l}\text{l}&=\left[\right(20.2\times \text{O}.\text{D}645+\left(8.02\times \text{O}.\text{D}663\right)]\\ &\quad\times \text{V}/1000\times \text{W}\end{aligned}$$

4 $$\begin{aligned} Carotenoids&=[O.D480+\left(0.114\times \text{O}.\text{D}663\right)]\\ &\quad\times (0.638\times \text{O}.\text{D}645)\end{aligned}$$

The approach of Barrs and Weatherley [23] was used to analyse the RWC of the leaves of *Zea mays*, *Amaranthus caudatus*, and *Medicago sativa*. A 100 mg samples of fresh ginger leaves were put in the Petri dishes and the water was applied to the dishes for 4 h. The water amount in leaves in *Zea mays*, *Amaranthus caudatus*, and *Medicago sativa* was measured after 4 h. The formula used for calculation is mentioned in Eq. 5.

5 $$\begin{aligned}\text{L}\text{e}\text{a}\text{f}\;\text{R}\text{e}\text{l}\text{a}\text{t}\text{i}\text{v}\text{e}\;\text{W}\text{a}\text{t}\text{e}\text{r}\;\text{C}\text{c}\text{o}\text{n}\text{t}\text{e}\text{n}\text{t}&=\frac{\text{f}\text{r}\text{e}\text{s}\text{h}\;\text{w}\text{e}\text{i}\text{g}\text{h}\text{t}-\text{d}\text{r}\text{y}\;\text{w}\text{e}\text{i}\text{g}\text{h}\text{t}}{Turgid\;weight-dry\;weight}\\&\quad\times 100 \end{aligned}$$

### Soil nutrient analysis

The soil’s agrochemical properties were evaluated after cultivation. The enhanced process was used to determine the levels of humus and carbon content, as per the GOST of the Commonwealth of Independent States from 2003. The standard method GOST 26261-84, GOST of Commonwealth of Independent States from 2005, and GOST of Commonwealth of Independent States from 2002 [24] were employed to analyze the total nitrogen, potassium, and phosphorus levels in the soil.

### Soil enzyme analysis

The soil’s urease activity was assessed using the methodology developed by Pansu and Gautheyrou [25]. Toluene (0.5 mL) was applied to soil samples (2.5 g) and kept for 15 min. Following the combination, 2.5mL of urea and the buffer (citrate) were introduced into 5mL—place in an incubator at 38 °C for 24 h. Spectrophotometer (405 nm) was employed to quantify urease activity. The soil enzyme activities, specifically catalase and invertase, were measured per the method developed by Khaziev [26]. Invertase activity (mg glucose kg^− 1^ h^− 1^) was determined by the determination of the glucose released in the hydrolysis reaction after incubation of samples with sucrose (8%) for 3 h at 37 °C. Soil catalase activity (mg KMnO_4_ kg^− 1^) was determined by measuring the reduction in H_2_O_2_ by titration with 0.1 M KMnO_4_ after having shaken a 5 g soil sample in 100 ml distilled water for 30 min.

### Quantitative analyses

The experimental data was analysed using SPSS V29. The recorded data were characterised using measures of central tendency (mean), and dispersion (standard deviation). Biochar impact on various plant species involved the use of one- and two-way ANOVA as well as MANOVA. To conduct more comparisons across groups, Duncan’s Multiple Range Test was employed at a significance level of 0.05. The significance of the treatment effect was judged via the magnitude of F-value (*p* < 0.05, < 0.01 as well as < 0.001).

### Statement on guidelines

The study was under relevant institutional, national, and international guidelines and legislation.

#### Permission Statement

Permissions or licenses were obtained for the collection of all seeds/plants.

## Results

Data shown in (Table 4) demonstrates the impact of food-biochar incorporation on the physical characteristics of *Zea mays*, *Amaranthus caudatus*, and *Medicago sativa* plants when exposed to high levels of salt. The salt stress considerably reduced the morphological characteristics of *Zea mays*, *Amaranthus caudatus*, and *Medicago sativa*.

**Table 4:** Impact of biochar on growth parameters of *Zea mays*, *Amaranthus caudatus*, and *Medicago sativa* under salt stress conditions

Plant	Treatments	Height of plant (cm)	Fresh weight of root (g)	Fresh weight of shoot (g)	Biomass allocation
*Medicago sativa*	Control	16.02 ± 1.61^d^	0.04 ± 0.01^e^	0.05 ± 0.01^e^	1.51 ± 0.34^a^
	Biochar	20.23 ± 0.10^b^	0.05 ± 0.01^d^	0.07 ± 0.02^c^	1.46 ± 0.04^a^
*Amaranthus caudatus*	Control	14.39 ± 1.49^e^	0.08 ± 0.01^b^	0.12 ± 0.01^b^	1.57 ± 0.09^a^
	Biochar	17.04 ± 0.91^cd^	0.11 ± 0.01^a^	0.18 ± 0.02^a^	1.81 ± 0.03^a^
*Zea mays*	Control	19.12 ± 1.01^c^	0.05 ± 0.01^d^	0.05 ± 0.01^e^	0.77 ± 0.03^b^
	Biochar	27.43 ± 0.52^a^	0.07 ± 0.01^c^	0.07 ± 0.02^d^	0.77 ± 0.02^b^

*Significant at *p* < 0.05, ** significant at *p* < 0.01, *** significant at *p* < 0.001; ns, non-significant at *p* > 0.05 according to a two-way analysis of variance (ANOVA): ^a, b^ Means followed through diverse letters vertically (in the same column) are expressively different rendering to Duncan’s multiple range tests (DMRTs).a

Biochar application has shown significant benefits on the growth of different plant species under salt stress conditions. For instance, *Medicago sativa*, showed a 29% increase in plant height, 34% enhancement in root’s fresh weight, and 51% increase in shoot fresh weight, compared to the control group. Similarly, the use of biochar for *Amaranthus caudatus* resulted in a 25% increase in plant height, 44% increase in root fresh weight and 55% increase in fresh mass of shoot, compared to the control group, under saline conditions. Furthermore, incorporating biochar into the sand has shown a substantial improvement in plant height by 49%, along with a 26% increase in fresh weight of shoot and 51% increase in fresh weight of root in *Zea mays* plants exposed to salt stress.

The data presented that the use of the food-biochar treatments had a considerable beneficial impact on root parameters and the ability to control salt stress, as indicated in Table 5. Under salt-induced stress conditions, the use of food-biochar treatment for *Medicago sativa* resulted in a considerable increase of 40% in root projected areas and 32% in root surface area, compared to the control group. The biochar addition greatly enhanced the root volume, roots diameter, and total length of the root by 83, 48, and 47% respectively, compared to the control. The use of biochar for *Amaranthus caudatus* resulted in a 25% rise in root surface area, a 26% surge in the projected region, and a 28% increase in diameter compared to the control group under salinity. Under a salt-induced environment, the application of biochar resulted in a substantial 37% increase in the volume of roots and a 51% increase in total root length. The biochar treatment resulted in significantly higher levels of root surface area (64%) in *Zea mays* than in the control samples. Under salt-induced conditions, the application of biochar to *Zea mays* plants leads to a considerable increase in root parameters than the control. Specifically, roots projected area by 33%, increases the total length of roots by 25%, the diameter of roots increased by 44%, and the volume of roots increased by 43%.

**Table 5:** Effect of food-biochar incorporation on the root characteristics of *Zea mays, Amaranthus caudatus*, and *Medicago sativa* in a condition of salt-induced stress

Plant	Treatments	Total root length (cm)	Root surface area (cm^2^)	Root projected area (cm^2^)	Root diameter (mm)	Root volume (cm^3^)
*Medicago sativa*	Control	16.21 ± 2.09^f^	6.71 ± 0.12^e^	1.83 ± 0.03^f^	0.69 ± 0.03^c^	0.12 ± 0.01^e^
	Biochar	23.24 ± 0.80^e^	8.63 ± 0.03^e^	2.63 ± 0.04^e^	1.02 ± 0.03^a^	0.18 ± 0.01^d^
*Amaranthus caudatus*	Control	39.81 ± 1.99^d^	17.84 ± 0.51^d^	6.12 ± 0.05^d^	0.90 ± 0.03^b^	0.55 ± 0.03^b^
	Biochar	59.11 ± 2^c^	21.64 ± 0.84^c^	7.93 ± 0.06^c^	1.14 ± 0.01^a^	0.69 ± 0.02^a^
*Zea mays*	Control	116.50 ± 3.04^b^	26.04 ± 0.13^b^	10.01 ± 0.52^b^	0.71 ± 0.04^c^	0.43 ± 0.04^c^
	Biochar	270.39 ± 2.07^a^	41 ± 0.27^a^	13 ± 0.22^a^	1.01 ± 0.02^b^	0.57 ± 0.02^b^

*Significant at *p* < 0.05, ** significant at *p* < 0.01, *** significant at *p* < 0.001; ns, non-significant at *p* > 0.05 according to two way ANOVA. a, b Means followed by different letters vertically (in the same column) are significantly different according to DMRTs

**Fig. 1:**
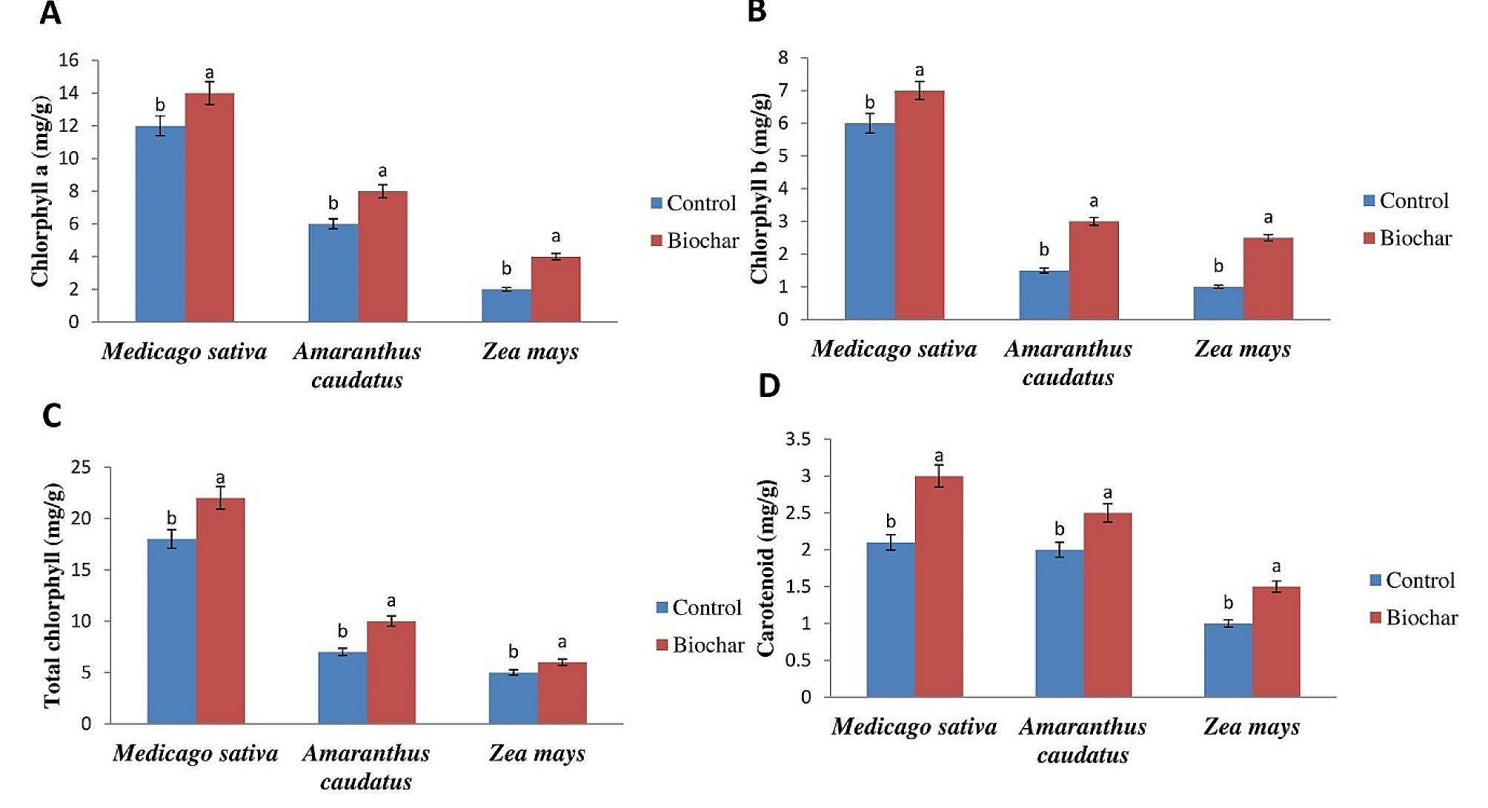
Effect of biochar application on photosynthetic pigments of *Zea mays*, *Amaranthus caudatus*, and *Medicago sativa* under salt stress. Bars labeled with distinct letters exhibit statistically significant differences as determined by DMRTs (Duncan’s Multiple Range Test) at a significance level of 0.05

The data demonstrated that the application of biochar resulted in an increase in photosynthetic pigments of *Zea mays*, *Amaranthus caudatus*, and *Medicago sativa* when compared to the control group that was subjected to salt stress (Fig. 1).

**Fig. 2:**
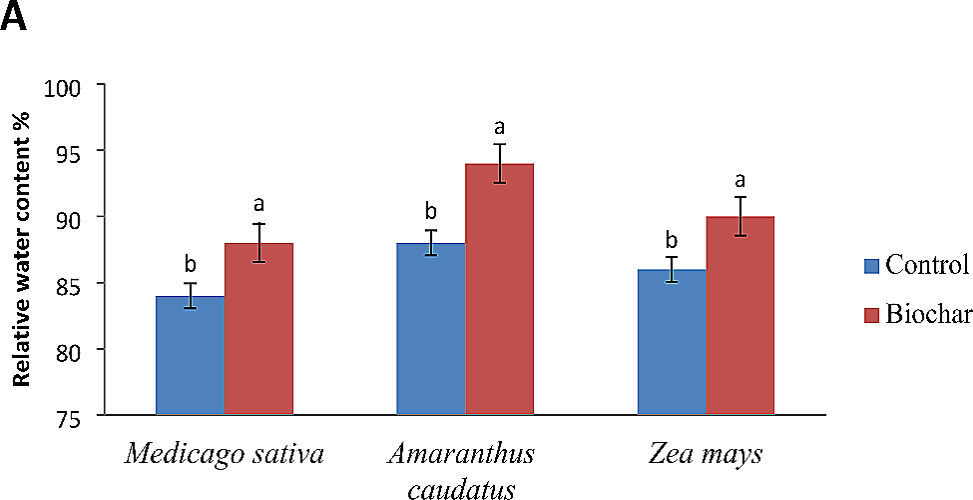
The impact of biochar on (**A**) the relative water content and (**B**) the biomass allocation (shoot: root ratio) of *Zea mays*, *Amaranthus caudatus*, and *Medicago sativa* under saline conditions. The bars that are followed by distinct letters exhibit substantial differences as determined by DMRTs (Duncan’s Multiple Range Test)

Application of biochar in *Medicago sativa* resulted in a considerable increase in chlorophyll a level by 9%, chlorophyll b level by 11%, the total amount of chlorophyll by 10%, and the content of carotenoid increased by 28% compared to the control group, under saline conditions. When *Amaranthus caudatus* was exposed to salt stress, the application of biochar resulted in a considerable increase of 40% in chlorophyll b pigments, 23% in total chlorophyll content, and 14% enhancement in carotenoid level compared to the control group. After biochar addition, *Zea mays* exhibited the greatest level of the chlorophyll-b, showing a notable increase of 66% compared to the control group, under salinity conditions. The data shown in Fig. 2 demonstrates that salinity has a negative impact on the relative water contents of leaves in *Zea mays*, *Amaranthus caudatus*, and *Medicago sativa*. Biochar treatments enhanced the relative water content of the leaves of *Zea mays, Amaranthus caudatus*, and *Medicago sativa* compared to the control. The highest relative water content was observed in *Amaranthus caudatus* after the application of biochar.

The data presented in (Table 6) demonstrates the significant influence of food-biochar incorporation on the soil’s nutrients under saline stress conditions. The application of the food-biochar enhanced the levels of soil’s nutrients including nitrogen, phosphorus, potassium, and humus levels. The application of biochar in *Medicago sativa* resulted in a considerable increase in Nitrogen level by 19%, phosphorus level by 17%, potassium level by 18%, and humus level by 56% compared to the control groups. The levels of nitrogen, phosphorus, potassium, and humus in *Amaranthus caudatus* showed a 16%, 18%, 18%, and 21% rise, respectively, after the application of biochar. Nevertheless, *Zea mays* had the greatest concentrations of potassium and phosphorus when biochar was applied. The application of biochar in *Zea mays* resulted in a significant increase in humus level (29%), potassium level (27%), and phosphorus level (25%).

**Table 6:** Impact of the food-biochar application on the soil’s nutrient levels under salinity stress

Plant	Treatments	Humus (%)	Potassium (mg/kg)	Phosphorus (mg/kg)	Nitrogen (mg/kg)
*Medicago sativa*	Control	0.08 ± 0.01^e^	51.31 ± 0.25^d^	5.23 ± 0.01^b^	4 ± 0.02^b^
	Biochar	0.15 ± 0.01^c^	53.27 ± 0.66^c^	6.60 ± 0.05^a^	5.01 ± 0.02^a^
*Amaranthus caudatus*	Control	0.11 ± 0.01^e^	56.2 ± 1.35^b^	3.02 ± 0.05^e^	3.21 ± 0.01^d^
	Biochar	0.13 ± 0.01^d^	66.8 ± 0.36^a^	3.56 ± 0.09^d^	3.96 ± 0.01^c^
*Zea mays*	Control	0.19 ± 0.01^b^	52.3 ± 2^cd^	3.41 ± 0.01^d^	2.76 ± 0.01^d^
	Biochar	0.24 ± 0.01^a^	67 ± 0.88^a^	3.81 ± 0.05^c^	2.97 ± 0.06^d^

*Significant at *p* < 0.05, ** significant at *p* < 0.01, *** significant at *p* < 0.001; ns, non-significant at *p* > 0.05 according to two way ANOVA. a, b Means followed by different letters vertically (in the same column) are significantly different according to DMRTs

The effects of food-biochar application on the enzymatic activity under salinity are presented in Table 7. Biochar application in *Medicago sativa* led to a considerable rise of 25% in urease function, 40% in invertase activity, and 30% in catalase activity in saline soil conditions. In *Amaranthus caudatus*, the addition of biochar to the soil under salt stress resulted in a 12% rise in catalase function and a 17% enhancement in urease activity than control. After the biochar addition, *Zea mays* exhibited a 13% rise in catalase function and a 24% rise in urease function under salt stress conditions.

**Table 7:** Impact of biochar application on soil enzymatic activity in conditions of salt stress

Plant	Treatments	Urease (NH_4_/g of soil/h)	Invertase (µgglucose·g ^−1^ soil·h ^−1^)	Catalase (mL KMnO4 g − 1 soil h − 1)
*Medicago sativa*	Control	2.37 ± 0.01^b^	2.61 ± 0.01^e^	3.09 ± 0.02^f^
	Biochar	3.90 ± 0.01^a^	3.17 ± 0.01^d^	3.94 ± 0.04^e^
*Amaranthus caudatus*	Control	2.30 ± 0.01^c^	6.21 ± 0.03^b^	8 ± 0.05^b^
	Biochar	3.51 ± 0.01^b^	7 ± 0.02^a^	8.97 ± 0.02^a^
*Zea mays*	Control	2.10 ± 0.01^d^	4.12 ± 0.01^c^	4 ± 0.02^d^
	Biochar	2.78 ± 0.01^b^	4.82 ± 0.01^c^	4.83 ± 0.02^c^

*Significant at *p* < 0.05, ** significant at *p* < 0.01, *** significant at *p* < 0.001; ns, non-significant at *p* > 0.05 according to two way ANOVA. a, b Means followed by different letters vertically (in the same column) are significantly different according to DMRTs

The thorough effects of food-biochar application on the growth parameters, enzyme activities, and physiological parameters under salinity are summarized in (Table 8). The table presents a multivariate exploration of the variance that examines the impact of food-biochar application, various plant species, and the relationship between biochar and plants. A significant overall impact of biochar was observed in various variables examined, such as plant height, root and shoot fresh weight, total root Projected Area, total length, root diameter and volume, total surface area, photosynthetic pigments, Urease, invertase and catalase, nitrogen, Phosphorus, potassium, and humus level.

**Table 8:** Multivariate exploration of variance was conducted to assess the effect of food-biochar treatments on several physiological attributes of different plants

Variables	Corrected-model		Plants		Treatments		Plant * Treatment	
	F	p-value	F	P-value	F	P-value	F	P-value
Plants height	72	< 0.01***	89.2	< 0.01***	149.1	< 0.01***	14.1	< 0.01***
Root fresh weight	67.9	< 0.01***	138.8	< 0.01***	59.2	< 0.01***	0.8	0.506^ns^
Shoot fresh weight	919.2	< 0.01***	1924.3	< 0.01***	551.3	< 0.01***	90.0	< 0.01***
Allocation of biomass	7.7	0.004**	16.7	< 0.01***	0.5	0.555^ns^	0.8	0.513^ns^
Total length	6362	< 0.01***	12601.2	< 0.01***	3489.7	< 0.01***	2249.2	< 0.01***
Total surface area	493.8	< 0.01***	1121.3	< 0.01***	218.6	< 0.01***	82.6	< 0.01***
Total projected area	576.3	< 0.01***	1473.2	< 0.01***	139.8	< 0.01***	24.1	< 0.01***
Root diameter	47	< 0.01***	25.9	< 0.01***	169.9	< 0.01***	1.5	0.273^ns^
Root volume	331.4	< 0.01***	691.2	< 0.01***	250.1	< 0.01***	7.1	0.012*
Total chlorophyll	1321.2	< 0.01***	3012.2	< 0.01***	53.9	< 0.01***	14.1	< 0.01***
Chlorophyll a	466.3	< 0.01***	1330.4	< 0.01***	20	< 0.01***	0.8	0.440^ns^
Chlorophyll b	831.2	< 0.01***	2110.1	< 0.01***	109.3	< 0.01***	1.3	0.291^ns^
Carotenoids	308.4	< 0.01***	701.6	< 0.01***	150.1	< 0.01***	14.2	< 0.01***
Relative water content	5.1	0.017*	9.4	0.003*	2.2	0.182^ns^	0.3	0.792^ns^
Urease	59.9	< 0.01***	37.9	< 0.01***	169.4	< 0.01***	24	< 0.01***
Invertase	230.1	< 0.01***	571.3	< 0.01***	21	< 0.01***	0.8	0.440^ns^
catalase	589.3	< 0.01***	1397.8	< 0.01***	70	< 0.01***	0.9	0.439^ns^
Potassium	244.1	< 0.01***	324.3	< 0.01***	446	< 0.01***	64.9	< 0.01***
Phosphorus	800.3	< 0.01***	1893.4	< 0.01***	180.3	< 0.01***	12	< 0.01***
Nitrogen	52.8	< 0.01***	106.3	< 0.01***	55	< 0.01***	0	> 0.999^ns^
Humus	27	< 0.01***	8.9	0.004**	97.1	< 0.01***	8.9	0.004**

*Significant at *p* < 0.05, ** significant at *p* < 0.01, *** significant at *p* < 0.001; ns, non-significant at *p* > 0.05 rendering to MANOVA

## Discussion

Salinity-induced stress is a prominent climatic element that restricts the growth of plants worldwide. It can result in a significant decrease in the height of plants, length of roots, fresh and dry weight of plants, and crop production [27]. In this work, salinity shown to have a negative impact on the amount of photosynthetic pigments in the leaves of *Zea mays*, *Amaranthus caudatus*, and *Medicago sativa* as shown in Fig. 1. Furthermore, the levels of total chlorophyll and chlorophyll a in *Solanum lycopersicum* plants observed a significant reduction [28]. The salinity conditions caused damage to the active photosynthetic activity in leaves, resulting in premature leaf senescence and chlorosis. This negative effect of salinity aligned aside with different research for instance, it was noted that *Zea mays* genotypes decrease in both chlorophyll and carotenoid content when exposed to salt stress [29]. Salt stress led to a significant decline in plant nutrients and photosynthetic pigments in both spring and winter wheat, as observed in other study [30]. This study has found that biochar enhanced photosynthetic pigments including carotenoid, and chlorophyll levels in *Zea mays*, *Amaranthus caudatus*, and *Medicago sativa*. This is consistent with research carried out by some scientists which shows that biochar can enhance nutrient uptake and improve the physiological traits of plants [28, 31] under salt stress. The use of biochar in this research has the potential to improve the physical attributes of the selected plants under salt stress. The study showed that the addition of biochar had a positive effect on the physiological properties of selected plants. The net photosynthesis pigments were significantly increased by biochar treatment alone. Biochar treatment also significantly increased the content of chlorophyll a, chlorophyll b, total chlorophyll, carotenoid, and relative water of the leaf over the control. Many researchers found that biochar application increased the photosynthesis, chlorophyll content, and transpiration rate in different plants [3]. However, the addition of biochar enhanced the photosynthetic activity of plants, especially at high concentrations of biochar, which may be due to a decrease in Na^+^ content followed by an increase in chloroplast ultrastructure. Our results similar to the findings of MK et al. [32], who reported that biochar incorporation, resulted in improved plant growth and root length in spinach under saline stress. The improvement of root morphology following biochar amendment and its contribution to enhanced salinity tolerance in plants can be attributed to various mechanisms. Here are some ways in which applied biochar may improve root morphology and subsequently help plants withstand salinity stress [11]. Biochar improves soil structure and porosity, providing a more favorable environment for root growth. Enhanced root length and surface area allow plants to explore a larger soil volume, facilitating greater nutrient and water uptake, which is crucial for salinity tolerance [18]. The potential advantages of biochar application on photosynthetic capacity, leaf area index, photosynthetic pigments, and transpiration rate had a substantial positive impact on the photosynthesis of leaves and the overall growth of rice as well as yield [33]. It is attributed to the biochar improves the biological nitrogen fixation processes by stimulating bacterial nitrification rates and increasing the nitrogen available for plant uptake. Biochar application also alleviates the adverse effects of abiotic stress on microorganisms [27]. The biochar addition improved significantly the salt resistance of cabbage seedlings and substantially augmented the levels of total chlorophyll, and chlorophyll-a, chlorophyll-b while reducing the levels of H_2_O_2_, proline, sucrose. The increase in photosynthetic pigment content under salinity stress following the application of biochar can be attributed to several mechanisms. While the specific effects may vary depending on the experimental conditions and plant species, here are some general ways in which biochar may contribute to higher photosynthetic pigment content in plants under salinity stress [13]. The biochar addition to saline soils improves stomata density and stomatal conductance, which improve leaf gas exchange characteristics, resulting in a substantial increase in photosynthetic efficiency under salt stress [23]. Biochar enhances nutrient availability in the soil, promoting the uptake of essential nutrients such as nitrogen, phosphorus, and potassium. These nutrients are crucial for chlorophyll synthesis, the primary photosynthetic pigment in plants [19]. The porous structure of biochar aids in improving soil water retention. Under salinity stress, maintaining adequate water availability is essential for photosynthesis. Enhanced water retention by biochar can alleviate water stress and support optimal photosynthetic activity [21]. Biochar’s ability to reduce oxidative stress may help protect the photosynthetic machinery and maintain higher levels of photosynthetic pigments; biochar may help maintain a more favorable ion balance, preventing the excessive accumulation of toxic ions because it can absorb harmful substances and mitigate their toxic effects on plants. This can contribute to the preservation of chlorophyll and other photosynthetic pigments [27]. Alfadil et al. [34] observed that adding biochar to soil affected by salinity stress greatly influenced *Zea mays* capacity to uptake nutrients and water retention. Biochar was found to alleviate osmotic stress by enhancing soil moisture and releasing mineral nutrients in both plants and soil solutions. This effect is attributed to biochar’s strong ability to bind with sodium ions, because of its high capacity for adsorption. Biochar can absorb more sodium ions into the soil. This process helps release nutrients and reduces osmotic stress via improving the water holding capacity of and increasing carbon storage. As a result, there is a significant improvement in photosynthetic activity, transcription rates, and stomatal conductance [35]. In addition, biochar effectively decreased the ratio of sodium and potassium ions and sodium content in numerous plants, thereby mitigating the adverse effects of salinity on the plants [36].

The application of biochar has the potential to directly enhance the nitrogen content of soils impacted by salt. Furthermore, it had an indirect impact on activity and bacterial abundance, possibly prompting the process of nitrogen transformation and promoting the release of nitrogen [37]. Furthermore, a significant elevation in the soil’s nitrogen content was observed, and this rise exhibited a strong correlation with the dosage of biochar that was applied. These findings validated the capacity of biochar to retain nutrients. The biochar treatment improved significantly the quantity of the soil organic matter. Biochar addition to extremely saline soil leads to significant enhancements in nutrient levels and soil organic matter. Several studies have indicated that biochar has the potential to serve as a direct supply of phosphorus and indirectly enhance soil texture, hence enhancing the phosphorus level in the soils impacted by salt [38]. Alfadil et al. [34] stated similar results, indicating that biochar enhanced nutrient levels in salt-stressed soil. The changes in soil nutrient content in response to different plant species and applied biochar can be influenced by a variety of factors, and the complexity of interactions in the soil-plant system plays a significant role. Here are some key reasons why soil nutrient content may change differently based on plant species and biochar application (i) biochar may influence the rhizosphere environment, further modulating nutrient interactions, (ii) Biochar application can also alter nutrient availability, impacting plant nutrient uptake differently based on their requirements, (iii) Microbes play a crucial role in nutrient cycling, and their responses to both plant roots and biochar can result in varying effects on soil nutrient content. Biochar amendments can influence microbial diversity and activity, impacting nutrient transformations, (iv) The surface area, porosity, and chemical composition of biochar can affect nutrient adsorption, desorption, and availability in the soil. These properties can lead to different nutrient dynamics depending on the biochar type [40]. According to Derbali et al. [39], biochar enhances soil organic carbon and augments the availability of nitrogen, phosphate, and potassium in under salinity. The application of biochar resulted in a 30% increase in SOC. The low degradability of the aromatic structure of carbon in biochar results in just a small portion, approximately 2%, of the biochar’s carbon being liberated from the soil as carbon dioxide by microbial respiration. Rezaie et al. [40] found that biochar enhanced the accessibility of soil total nitrogen, phosphorus, and potassium in saline environments. Ramzani et al. [41] have reported similar findings. Our findings suggested that - addition of biochar enhanced the levels of total phosphorus and nitrogen as well as soil organic matter under saline conditions. It is attributed that biochar application to soil may directly or indirectly influence soil nutrient dynamics via a range of mechanisms including altering soil pH, stimulating the formation of organo-mineral complexes or altering P adsorption/desorption equilibrium, altering P solubility by influencing microbial enzyme activities. In contrast to P, biochar additions to soil have been found to induce either positive, negative, or neutral effects on soil inorganic N availability and the mechanisms driving these changes have been argued to be both abiotic (such as adsorption or desorption) associated with N transformation processes (i.e. mineralization, immobilization, nitrification, fixation) [11]. Research carried out by Ma et al. [42], found that biochar enhanced the alkaline phosphatase and catalase activities of soil under salt stress environments. The improved nutrient status can positively influence root development and nutrient uptake. Adequate nutrient supply is essential for plant growth and can contribute to salinity tolerance [14].

The activity of soil microorganisms has been shown through the observation of enzymatic activity, which is dependent on alterations in the soil system [1] Beneficial microbes fostered by biochar may form symbiotic relationships with plant roots; promoting nutrient cycling and helping plants cope with environmental stresses, including salinity [5]. Furthermore, the activities of the soil enzymes play a crucial role in the degradation of soil organic substances and nutrient cycling [43]. These enzyme activities are also regarded as significant factors in soil health. Soil enzymes are considered strong indicators of soil status and fertility as their activities are related to soil type, quality, and physiochemical properties. Nevertheless, there is a scarcity of exploration that specifically scrutinizes the effect of biochar on enzymatic activities in saline soils. The findings of our research suggested that biochar incorporation had a beneficial influence on enzyme activities in saline soil. The soil enzymatic activities of urease, and soil catalase were significantly increased under biochar application compared to control. In the present study, the biochar effect was significant on all enzymes; in contrast, the nitrogen effect was only effective on urease and negatively affected the activities of other enzymes, which indicates the positive and synergetic effect of biochar in promoting soil quality. The positive response of urease to biochar addition is crucial as this enzyme takes part in cycling and availability of N [42] and this high activity of urease in the presence of biochar might be due to the high N mineralization and retention effect of biochar. These results suggest that biochar can more efficiently promote the hydrolysis of applied nitrogen fertilizers. The transformation of the soil’s organic nitrogen into available inorganic-nitrogen was associated with the enzyme urease. In the process of soil improvement, enzymes like invertase and catalase play a crucial role. Invertase helps to increase the amount of soluble nutrients in the soil which in turn provides energy to the microorganisms present in it. Catalase helps in the oxidation of organic matter and the production of humus. It also affects the microbial activity and biological redox in the soil. The application of biochar in the current experiment had a significant impact on the activities of urease, invertase, and catalase, compared to the control treatments.

These results suggest that biochar application may develop cellular redox homeostasis by stimulating the ability of antioxidants to scavenge ROS generated by salt, thereby improving plant salt tolerance. Biochar utilization has the potential to augment enzymatic activity through the enhancement of soil organic matter, microbial biomass, and activity. In addition, it can facilitate enzyme interaction by putting them into proximity with biochar surface [39]. The improvement of soil enzyme activities under salinity conditions due to added biochar can be attributed to various mechanisms. Soil enzymes play a crucial role in nutrient cycling, organic matter decomposition, and overall soil health. Here’s how biochar may enhance soil enzyme activities under salinity stress and contribute to improved plant resilience [31]. Biochar provides a favorable habitat for soil microorganisms. The increased microbial activity, facilitated by biochar, can contribute to higher enzyme production. Microbes play a key role in producing extracellular enzymes that participate in nutrient cycling processes [37]. Biochar can help in mitigating the toxic effects of salts. By reducing ion imbalances and toxicity, biochar promotes a healthier microbial community. Healthy microbial populations are more likely to produce and secrete enzymes that contribute to nutrient cycling [27]. Enhanced soil enzyme activities contribute to efficient nutrient cycling. This can be particularly crucial under salinity stress when nutrient uptake by plants may be compromised. Improved nutrient availability supports plant growth and salinity tolerance [18]. Soil enzymes involved in organic matter decomposition release essential nutrients. These nutrients become available to plants, promoting their overall health and resilience to salinity stress [20]. The findings of these positive impacts on soil physiochemical properties, enzymatic activities, and plant growth attributes indicate that biochar amendment had improved soil conditions, plant physiological attributes, and plant growth.

## Conclusion

Salinity stress adversely impacted the growth, root morphology, and photosynthetic pigments of *Zea mays, Amaranthus caudatus*, and *Medicago sativa*. Salinity decreased the levels of chlorophyll a and b, as well as carotenoids in *Zea mays, Amaranthus caudatus*, and *Medicago sativa*. Biochar treatments enhance significantly root morphological features and plant growth metrics of *Zea mays, Amaranthus caudatus*, and *Medicago sativa* under salt stress than the control group. When exposed to salt stress, using biochar can enhance photosynthetic pigments such as total chlorophyll concentration, chlorophyll a, and chlorophyll b. Biochar treatments enhanced nitrogen, phosphorus, potassium, and humus levels in the soil under salt stress conditions. Biochar treatments stimulate soil enzyme activity including catalase, invertase, and urease, promoting nutrient availability and reducing the need for chemical fertilizers in salt-stressed conditions.

It has been suggested that using biochar in combination with microbes can help increase a plant’s ability to handle salt-induced stress. However, more research is needed to confirm the effectiveness of using biochar and microbes to enhance growth in the presence of salty conditions. It is also important to conduct pilot studies to determine the appropriate rates of biochar application, considering factors such as soil, plant, and climate conditions. To ensure sustainable agriculture in the future, it is essential to consider the combined impacts of multiple environmental factors on crop growth.

## Data Availability

The datasets analysed during this study are included in this manuscript.

## Declarations

### Statement on guidelines

All experimental studies and experimental materials involved in this research are in full compliance with relevant institutional, national and international guidelines and legislation.
